# The SARS-CoV-2 Infection Among Students in the University of Porto: A Cross-Sectional Study

**DOI:** 10.3389/ijph.2022.1604548

**Published:** 2022-10-20

**Authors:** Paula Meireles, Joana Pinto Costa, Maria João Novais, Daniela Miranda, Mariana Mendes Lopes, Milton Severo, Henrique Barros

**Affiliations:** ^1^ EPIUnit, Instituto de Saúde Pública da Universidade do Porto, Porto, Portugal; ^2^ Laboratório para a Investigação Integrativa e Translacional em Saúde Populacional (ITR), Instituto de Saúde Pública da Universidade do Porto, Porto, Portugal; ^3^ Departamento de Ciências da Saúde Pública e Forenses e Educação Médica, Faculdade de Medicina, Universidade do Porto, Porto, Portugal

**Keywords:** seroprevalence, university students, SARS-CoV-2, antibodies, seroepidemiology

## Abstract

**Objective:** We aimed to quantify SARS-CoV-2 specific antibodies’ seroprevalence among university students in Porto.

**Methods:** A rapid point of care testing for SARS-CoV-2 specific immunoglobulin (Ig) M and IgG antibodies was performed, and a questionnaire was applied to 6512 voluntary students from September to December 2020. We computed the apparent IgM, IgG, and IgM or IgG prevalence, and the true prevalence and 95% credible intervals (95% CI) using Bayesian inference.

**Results:** We found an apparent prevalence (IgM or IgG) of 9.7%, the true prevalence being 7.9% (95% CI 4.9–11.1). Prevalence was significantly higher among males (10.9% vs. 9.2%), international students (18.1% vs. 10.4% local vs. 8.8% nationally displaced), and increased with age. Those with a known risk contact, that experienced quarantine, had symptoms, or a previous negative molecular test had a higher seroprevalence. Of the 91 (1.4%) students who reported a molecular diagnosis, 86.8% were reactive for IgM or IgG.

**Conclusion:** Based on immunological evidence infection was 5.6-fold the reported molecular diagnosis. The higher seroprevalence among male, older, and international students emphasizes the importance of identifying particular groups.

## Introduction

Infection with the severe acute respiratory syndrome coronavirus 2 (SARS-CoV-2) can follow many distinct courses, with poor outcomes occurring mostly in the elderly population and no or few unspecific symptoms occurring mainly among young and healthy individuals [1–3]. Real-time Polymerase Chain Reaction (RT-PCR) is the diagnostic “reference standard”, but testing strategies changed over the course of the epidemic and varied according to local logistic capacity. Thus, confirmed cases are a suboptimal indicator of the extent of SARS-CoV-2 infection, and the magnitude of undiagnosed infections can vary widely [4]. SARS-CoV-2 seroprevalence studies are critical to monitor the epidemic evolution in a population and to inform public health measures, such as vaccine allocation [5]. Those studies estimate the number of past infections higher than the number of RT-PCR confirmed cases [6, 7]. In the case of an emergent agent, it is assumed that all population is initially susceptible; therefore, the presence of specific antibodies provides good estimates of the cumulative incidence particularly if the infection provides long-term serological immunity.

In Portugal, the first case of coronavirus disease 2019 (COVID-19) was diagnosed on March 2, 2020, and on March 16 a nationwide schools closure was decreed affecting all education levels [8]—around 2 million students, more than 346 thousand from higher education [9], were moved to remote teaching. The schools’ closure accompanied the implementation of even more restrictive non-pharmaceutical measures, such as lockdown, later eased over the 2020 summer months. Schools and universities resumed in-person teaching activities around mid-September 2020, providing an excellent opportunity to obtain data on the serum status of a large sample of university young adults exposed to highly varied risk contexts.

This study aimed to estimate SARS-CoV-2 specific antibodies’ seroprevalence and its determinants among students at the University of Porto (U.Porto), assessed between September and December of 2020.

## Methods

All undergraduate and postgraduate students from the U.Porto were sent an email by the University communication office to invite them to perform a rapid serological test for SARS-CoV-2 specific immunoglobulin (Ig) M and IgG antibodies. Along with this email, an information leaflet was sent, and the initiative was disseminated through the U.Porto online social networks. Participation was voluntary, and students scheduled their appointment according to their convenience. They were invited to answer a face-to-face questionnaire conducted by the trained researcher who performed the test while waiting for the result.

The questionnaire included the following demographic and social questions: sex, age, living in usual residence (yes; no, usual residence in the country; no, usual residence abroad), faculty, history of contacts with a confirmed SARS-CoV-2 case since January 2020, history of being quarantined since January 2020, symptoms (then categorized as asymptomatic; paucisymptomatic: defined as having or having had one or two of the following symptoms: cough, dyspnea, odynophagia, headache, vomiting or nausea, diarrhea, fever, arthralgias, myalgia, asthenia; and symptomatic defined as having or having had at least three symptoms listed before, or dysgeusia or anosmia), ever being tested for SARS-CoV-2 infection, previous SARS-CoV-2 infection diagnosis, dates of diagnosis and recovery, self-perception of the probability of having been infected (the English version of the questionnaire is available in Supplementary File S1).

Data reported in this study refer to the period between 24 September and 15 December 2020, during which 6512 students (approximately 20% of the 32,443 students of U.Porto) self-selected to have a point of care serological test. The participants’ characteristics are presented in Table 1.

**TABLE 1:** Description of the characteristics of the University of Porto students evaluated from September to December 2020, Porto, Portugal (Portugal, 2020).

Characteristics of the students	Total of participants	
	N	(%)
Overall	6512	(100.0)
Sex		
Female	4554	(69.9)
Male	1951	(30.0)
Missing	7	(0.1)
Age strata (years)		
<20	1600	(24.6)
20–24	3548	(54.5)
25–29	735	(11.3)
30–34	319	(4.9)
35–39	127	(2.0)
≥40	180	(2.8)
Missing	3	(0.0)
Living in usual residency		
Yes	3689	(56.6)
No, but usual residence in the country	2192	(33.7)
No, usual residence abroad	626	(9.6)
Missing	5	(0.1)
Confirmed case contact		
No	5624	(86.4)
Yes	878	(13.5)
Missing	10	(0.2)
Quarantined		
No	5757	(88.4)
Yes	748	(11.5)
Missing	7	(0.1)
Symptoms since January 2020		
Asymptomatic	4871	(74.8)
Paucisymptomatic^a^	689	(10.6)
Symptomatic^a^	947	(14.5)
Missing	5	(0.1)
Previous RT-PCR test and diagnosis		
Never tested	5062	(77.7)
Tested, RT-PCR negative	1358	(20.9)
Tested, RT-PCR positive	91	(1.4)
Missing	1	(0.0)
Self-perception of the probability of having already been infected (excluding those with diagnosis; *n* = 6421)		
Very low	860	(13.2)
Low	3134	(48.1)
Moderate	1954	(30.0)
High	351	(5.4)
Very high	117	(1.8)
Missing	5	(0.1)
Time since the diagnosis (among those previously diagnosed; *n* = 91)		
<2 months	59	(64.8)
2–5 months	13	(14.3)
≥6 months	19	(20.9)
Missing	0	(0.0)

^a^ Paucisymptomatic: having or having had one or two of the following symptoms: cough, dyspnea, odynophagia, headache, vomiting or nausea, diarrhea, fever, arthralgias, myalgia, asthenia; Symptomatic defined as having or having had at least three symptoms listed before, or dysgeusia or anosmia.

The study protocol was approved by the ethics committee of the Institute of Public Health of the University of Porto (ID 20154) and procedures were in accordance with the 1964 Helsinki declaration and its later amendments or comparable ethical standards. Verbal informed consent was obtained prior to the interview. Questionnaires were anonymous, and the results were only communicated to the students. The identifying information needed to schedule testing was kept only at the U.Porto information systems department. The linkage between datasets is impossible.

### SARS-CoV-2 Specific IgM and IgG Antibodies Determination

Three point-of-care tests were used according to the manufacturer instructions—the STANDARD Q COVID-19 IgM/IgG Combo (manufacturer reported sensitivity of 94.5% seven or more days after symptom onset and specificity of 95.7% for both IgG and IgM), the HIGHTOP—SARS-CoV-2 IgM/IgG Test Combo (manufacturer reported sensitivity of 82.0% and 93.0% and specificity of 96.0% and 97.5% for IgM and IgG, respectively), and the *Teste Rápido Pantest de Coronavirus 2019-nCoV IgG/IgM* (manufacturer reported sensitivity of 85.0% and 100% and specificity of 96.0% and 98.0% for IgM and IgG, respectively). The three manufacturers used RT-PCR as the gold standard. The first was used from 24 September to October 19 (*n* = 2263), the second from October 19 to 26 (*n* = 1059), and the third from 27 October onwards (*n* = 3190).

All participants presenting with symptoms or reporting high-risk contacts in the previous 14 days were recommended to contact the National Health Service Contact Centre. All participants were communicated their results orally and also in the form of a written leaflet with the information that the serological test only indicates whether there is evidence of previous contact with the SARS-CoV-2 and that it cannot be used to diagnose or rollout SARS-CoV-2 infection. It also recommended that all SARS-CoV-2 preventive measures were to be adopted and to call the National Health Service Contact Centre in case of symptoms.

### Statistical Analysis

We estimated seroprevalence as the proportion of individuals who had a reactive result in the IgM or IgG band of the point-of-care test. We estimated the true prevalence and 95% credible intervals (95% CI) using Bayesian inference. We used a uniform prior distribution for sensitivity ranging from 0.82 to 1 and specificity between 0.94 and 1. Estimates were obtained using the “rjags” package in R.

Groups were compared using the Pearson Chi-Square, or the Fisher-exact test when the chi-square test’s assumptions did not hold.

## Results

Table 2 presents the IgM, IgG, and IgM or IgG apparent seroprevalence and reported SARS-CoV-2 infection prior diagnosis by a molecular test according to the characteristics of the U.Porto students. Among the 6512 students evaluated, 558 (8.6%) had a reactive test for IgM, 380 (5.8%) for IgG, and 634 (9.7%) for IgM or IgG. The estimated true prevalence was 6.6 (95% CI 3.6–9.6) for IgM, 3.5 (95% CI 0.5–6.5) for IgG and 7.9 (95% CI 4.9–11.1) for IgM or IgG.

**TABLE 2:** The IgM, IgG and IgM or IgG apparent seroprevalence and reported SARS-CoV-2 infection diagnosis according to the characteristics of the University of Porto students evaluated from September to December 2020, Porto, Portugal (Portugal, 2020).

Characteristics of the students	Seroprevalence			Prior SARS-CoV-2 infection diagnosis
	IgM	IgG	IgM or IgG	
			N (%)	
Overall	558 (8.6)	380 (5.8)	634 (9.7)	91 (1.4)
Sex				
Female	370 (8.1)	241 (5.3)	421 (9.2)	62 (1.4)
Male	187 (9.6)	138 (7.1)	212 (10.9)	28 (1.4)
*p-value*	*0.060*	*0.006*	*0.048*	*0.907*
Age strata (years)				
<20	108 (6.8)	68 (4.3)	126 (7.9)	11 (0.7)
20–24	307 (8.7)	201 (5.7)	345 (9.7)	54 (1.5)
25–29	74 (10.1)	53 (7.2)	80 (10.9)	12 (1.6)
30–34	34 (10.7)	32 (10.0)	43 (13.5)	8 (2.5)
35–39	9 (7.1)	7 (5.5)	11 (8.7)	2 (1.6)
≥40	26 (14.4)	19 (10.6)	29 (16.1)	4 (2.2)
*p-value*	*0.002*	*<0.001*	*0.001*	*0.028*
Living in usual residency				
Yes	285 (7.7)	188 (5.1)	382 (10.4)	46 (1.2)
No, but usual residence in the country	174 (7.9)	102 (4.7)	193 (8.8)	21 (1.0)
No, usual residence abroad	99 (15.8)	90 (14.4)	113 (18.1)	24 (3.8)
*p-value*	*<0.001*	*<0.001*	*<0.001*	*<0.001*
Confirmed case contact				
No	401 (7.1)	227 (4.0)	452 (8.0)	27 (0.5)
Yes	156 (17.8)	152 (17.3)	180 (20.5)	64 (7.3)
*p-value*	*<0.001*	*<0.001*	*<0.001*	*<0.001*
Quarantined				
No	416 (7.2)	250 (4.3)	475 (8.3)	24 (0.4)
Yes	142 (19.0)	130 (17.4)	159 (21.3)	67 (9.0)
*p-value*	*<0.001*	*<0.001*	*<0.001*	*<0.001*
Symptoms since January 2020				
Asymptomatic	320 (6.6)	178 (3.7)	364 (7.5)	19 (0.4)
Paucissymptomatic^a^	68 (9.9)	45 (6.5)	72 (10.4)	10 (1.5)
Symptomatic^a^	166 (17.5)	155 (16.4)	194 (20.5)	60 (6.3)
*p-value*	*<0.001*	*<0.001*	*<0.001*	*<0.001*

^a^ Paucisymptomatic: having or having had one or two of the following symptoms: cough, dyspnea, odynophagia, headache, vomiting or nausea, diarrhea, fever, arthralgias, myalgia, asthenia; Symptomatic defined as having or having had at least three symptoms listed before, or dysgeusia or anosmia.

Italic values represent the discriminate *p*-values from the other values.

The prevalence of IgG was higher among males (7.1%) than among females (5.3%, *p* = 0.006). The prevalence of IgM or IgG antibodies was higher among the 30–34 years old and the 40 and more years old, 13.5% and 16.1%, respectively (vs. 7.9% in students under 20, 9.7% in 20–24 years, 10.9% in 25–29 years and 8.7% in 35–39 years, *p* = 0.001). A history of prior diagnosis was also higher in those age groups, 2.5% among the 30–34 years old and 2.2% in those aged 40 years or over (vs. 0.7% in students under 20, 1.5% in 20–24 years, 1.6% in 25–29 years and 1.6% in 35–39 years, *p* = 0.028).

The prevalence of antibodies was higher among international students (18.1% for IgM or IgG vs. 10.4% among those living in their family household and 8.8% among nationally displaced, *p* < 0.001). Also, the proportion reporting a previous RT-PCR infection diagnosis was higher among international students (3.8% vs. 1.2% among those living in their family household and 1.0% among nationally displaced, *p* < 0.001).

Students who had contact with confirmed cases showed a prevalence of IgM or IgG of 20.5%, higher than the prevalence of 8.0% among those without (*p* < 0.001). Similar results were found among those who were quarantined (21.3% vs. 8.3%, *p* < 0.001). IgM or IgG prevalence was also higher whenever there was a history of symptoms since the beginning of 2020, being 7.5% among asymptomatic, 10.4% among paucisymptomatic, and 20.5% among ever symptomatic students (*p* < 0.001).

**FIGURE 1:**
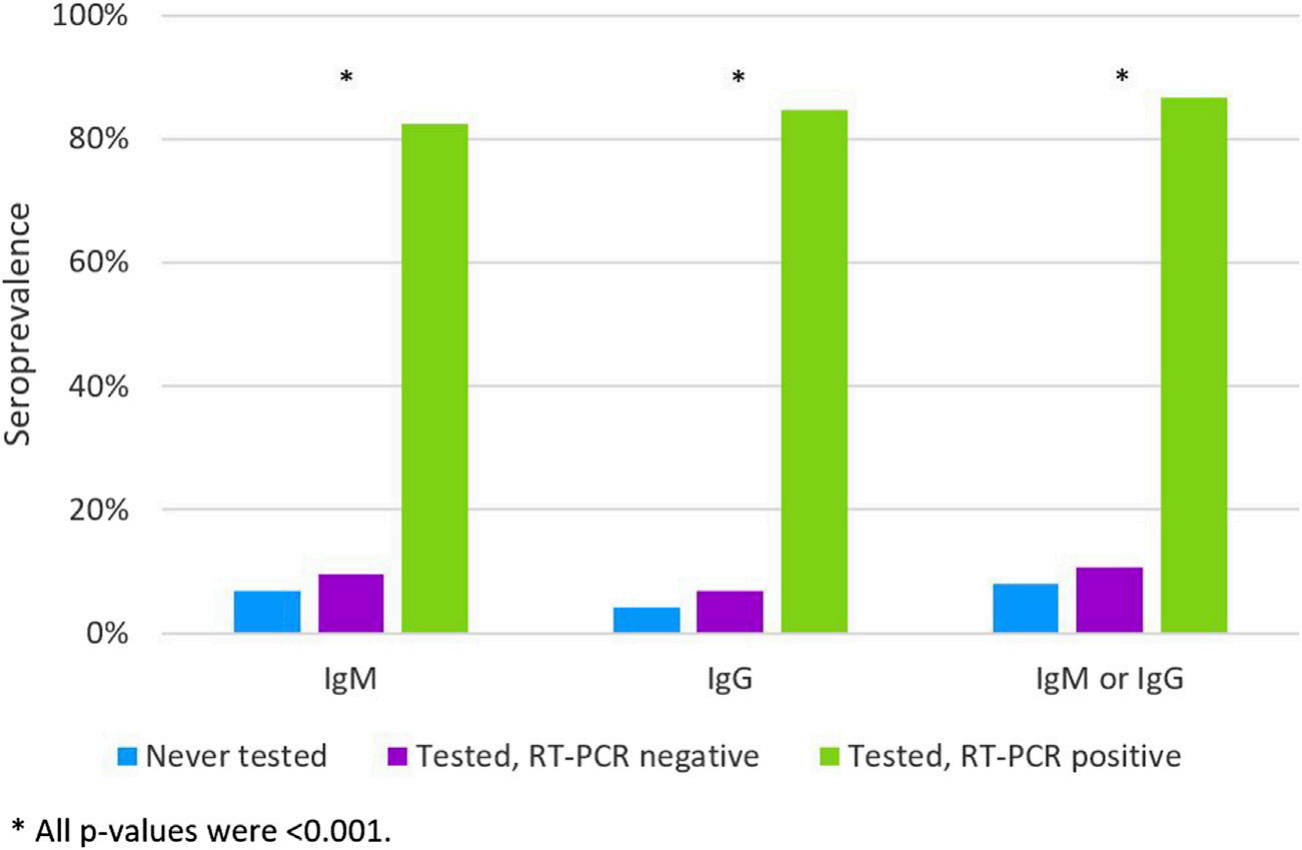
The IgM, IgG and IgM or IgG apparent apparent seroprevalence according to the history of previous RT-PCR test and diagnosis (Never tested; Tested, RT-PCR negative; Tested, RT-PCR positive) (*n* = 6512). (Portugal, 2020).

The SARS-CoV-2 infection had been previously diagnosed by a molecular test in 91 (1.4%) students. They had a prevalence of IgM or IgG antibodies of 86.8%, this prevalence was 10.7% in those who had a RT-PCR negative test and 8.1% in those never tested (*p* < 0.001) (Figure 1).

**FIGURE 2:**
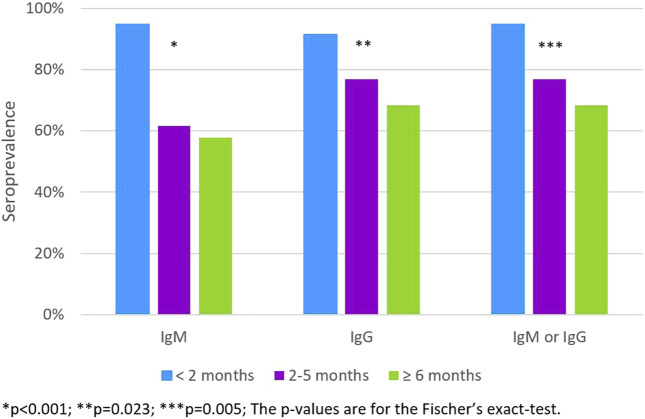
The IgM, IgG and IgM or IgG apparent seroprevalence according to the time since the RT-PCR diagnosis (<2 months; 2–5 months; ≥ 6 months) (*n* = 91). (Portugal, 2020).

Of the 91 (1.4%) students who had been previously diagnosed with SARS-CoV-2 infection, the prevalence of antibodies decreased with the increasing time since diagnosis, 76.9% among those diagnosed between two and 5 months and 68.4% among those diagnosed six or more months before the serological test (Figure 2).

**FIGURE 3:**
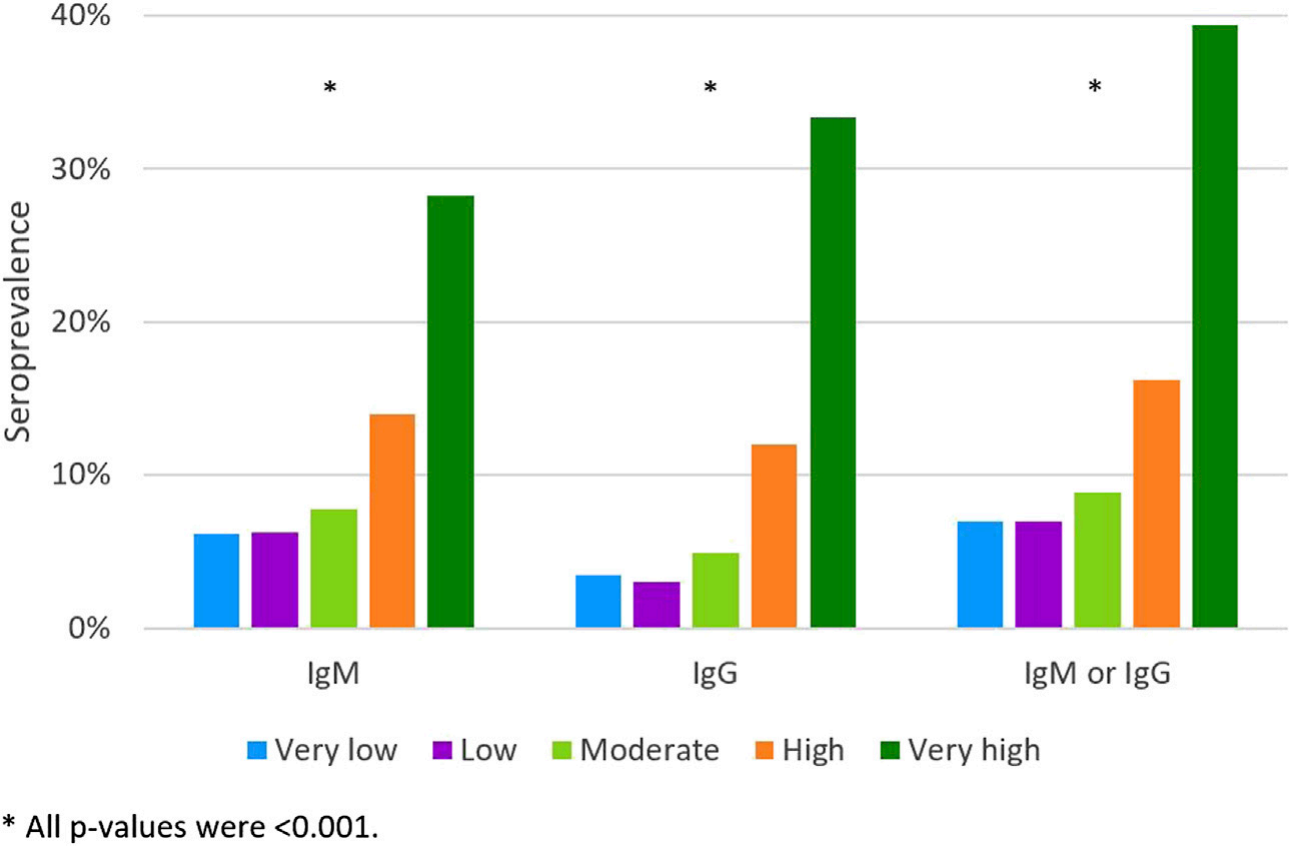
The IgM, IgG and IgM or IgG apparent seroprevalence according to the self-perception of the probability of having already been infected, excluding those with a previous RT-PCR diagnosis (Very low; Low; Moderate; High; Very high) (*n* = 6421). (Portugal, 2020).

Among students without an RT-PCR diagnosis of SARS-CoV-2 infection, the prevalence of antibodies increased with the increased perception of having been infected; it varied from 39.3% among those who considered this probability to be very high to 7.0% among those who thought it was low or very low (Figure 3).

## Discussion

The 6512 students had a 9.7% prevalence of IgM or IgG antibodies. However, only 1.4% reported a prior diagnosis of SARS-CoV-2 infection based on an RT-PCR result. The burden of infection in this group was 6.9 times higher than the reported cases considering the point estimate or 5.6 times higher if compared with the estimated true prevalence of 7.9%, as observed in previously published surveys [4, 6, 7, 10]. The lower true prevalence was expected. Even using high specificity and sensitivity tests there is a high number of false positives due to the relatively low frequency of infection in this population [11].

Students had a higher prevalence of infection than observed in the Portuguese serological survey (ISNCOVID-19), conducted between May and July 2020 (2.9%) [12]. Considering the participants in the age group 20–39 years the prevalence was 2.9% in the ISNCOVID-19 and 10.1% in the U.Porto students. However, the studies were conducted in different periods of the epidemic in Portugal. The cumulative incidence of notified SARS-CoV-2 infection at the end of the national survey was 0.4% while at the end of this study it was 3.5% [13]. Differences in the recruitment of participants and the population’s characteristics partially contribute to explain the observed differences in the prevalence.

Students were evaluated just after the beginning of the academic year 2020/2021, for which the recommendations were that in-person activities should be guaranteed, remote classes occurred only when safety measures could not be ensured. To reduce the risk of infection in the university context there were several non-pharmaceutical measures in place, such as self-surveillance of signs and symptoms and strict school, work or social eviction in the presence of symptoms suggestive of COVID-19, reduced capacity of spaces in order to ensure physical distance between people and to minimize contact with respiratory droplets, the mandatory use of masks in all university spaces, and recommendations for respiratory etiquette measures. Measures related to the reduction of environmental risk such as sanitation and ventilation of spaces were also in place. Therefore, these results probably reflect more the infection transmission in the community than in the university context.

Male and female students reported the same proportion of molecular diagnoses (1.4%). However, we found a higher seroprevalence among males, as reported in American university students [14], the Portuguese population [12] but not in other population-based surveys [4, 6, 7], and a meta-analysis [15]. We have no information on the study level (undergraduate or graduate) and therefore could not measure seroprevalence according to this variable, but older students had a higher seroprevalence. A previous study showed no difference in the prevalence of IgG antibodies in undergraduates and graduates suggesting they may have not had different lifestyles that would make them more or less susceptible [14] but considering age this may not have been the case in U.Porto. Students whose usual residence was abroad had a higher seroprevalence of infection. This might reflect a higher risk experience in their own countries or sharing a more vulnerable context during their stay in U.Porto.

We found higher seroprevalence among students who reported previous negative molecular test compared with those never tested, suggesting that some RT-PCR results might have been false negatives [16]. In accordance with previous studies, the self-reported belief of having had SARS-CoV-2 infection, prior contact with confirmed cases, and having had symptoms were positively associated with a higher seroprevalence [7, 12, 14].

Despite a previous RT-PCR positive test, 17.6% showed no IgM and 13.2% no IgG antibodies. These may be false-negative results, evidence of no immune response, or more likely waning of antibodies over time. The observed decreasing seroprevalence with increasing time after the diagnosis also supports this explanation, as previously described [17, 18]. However, it is important to note that more than two-thirds of RT-PCR positive students had detectable immunological evidence of infection more than 6 months after diagnosis, indicating that antibodies may last long in a substantial proportion of individuals, as previously reported [19].

The national cumulative incidence of notified COVID-19 cases was 3.5% by the end of our data collection. However, only 1.4% of students reported a positive RT-PCR test for SARS-CoV-2. This lower incidence may be partially explained by the higher proportion of young people with few or no symptoms and, therefore, unnoticed infections, students’ higher socioeconomic status, and an increased commitment to non-pharmacological preventive measures [1, 2]. It is worth mentioning that the number of reported cases among U.Porto students from 16 September 2020, to 16 December 2020, was 879, corresponding to 26 per 1000 students. This is almost double the observed in our sample and can have several explanations: 1. from an individual point of view those with a previous infection may have less interest in doing the serological test because they know already they had contact with the virus; 2. they may be enrolled in care or having already an antibody test provided by the clinical services, and 3. some of those infections were recent and therefore students may have not yet had the opportunity to perform the serological test as part of our survey. We observed an increasing number of reported infection diagnosis in our study since August, as observed nationwide, but only one case in November, which might indicate that those diagnosed more recently did not yet have the chance to participate in this serological testing program.

We used three different point-of-care tests over three different periods. This was unintended and was due to manufacturer delay on delivery which are constraints of real-world research in a time of high demand. However, all tests presented similar manufacturer’s reported characteristics, were used by the same trained researchers, and had similar performance in an in-house pilot test (data not shown). We cannot infer from this large sample to the U.Porto students’ population due to the non-probabilistic nature of the sample and the 20% participation rate. This participation rate may have several explanations: many students remained in remote learning, the recruitment strategy as students may have dismiss the invitation email due to frequently receiving institutional emails, the voluntary nature of this testing program, and due to the distance to the localization of the testing place located at the city centre while U.Porto has multiple poles distributed in the town. Compared to the eligible population (data not shown), our sample had a similar age distribution but a higher proportion of females (70% vs. 55%) and national students (90% vs 86%), which might result in the underestimation the seroprevalence of SARS-CoV-2 specific antibodies. Beyond these basic sociodemographic characteristics, the self-selection nature of recruitment may have also lead to selection bias by over capturing those who have had symptoms, high-risk contacts, or a self-perception of a higher probability of having been infected; those with a previous RT-PCR diagnosis appear to be underrepresented, making difficult to predict the direction of the bias. However, the magnitude of serological evidence of infection in such an educated, probably relatively low-risk community, is strong evidence of an increasing burden of COVID-19 in Portugal.

### Conclusion

At the University of Porto, students had an estimated true seroprevalence of 7.9%, five times higher than the prevalence based on the self-reported molecular diagnoses and two times higher than the notified national cumulative incidence by the end of the study. Being an international student, reporting symptoms, self-perceiving high probability of infection, having had contact with a case, experiencing quarantine, and having had a diagnostic RT-PCR test performed though negative was associated with higher seroprevalence. Antibodies were present in 87% of those previously diagnosed with a molecular test, though reactivity decreased with time since the diagnosis.
