# Supplementary material for: The SARS-CoV-2 Infection Among Students in the University of Porto: A Cross-Sectional Study
Source: Int J Public Health. 2022 Oct 20;67:1604548. doi: 10.3389/ijph.2022.1604548 (PMC9630354; doi:10.3389/ijph.2022.1604548)
Supplement: Supplementary file 1 [file DataSheet1.docx]

## **QUESTIONNAIRE**

**Screening technician ID:** |___|___|

**Date of screening:** |___|___| - |___|___| - |_____|_____|_ ___|_____|

**Time:** |___|___|H |___|___|MIN

# **SOCIODEMOGRAPHIC INFORMATION**

**P1: Sex:**

□ Male

□ Female

**P2. Age in years**? ____

**P3. Are you displaced? (**consider displaced if you are living outside your usual family residence**)**

If yes:

P3.1 From where?

□ Within the country

□ Outside the country

**P4. What is your organic unit?**

□ Faculty of Architecture (FAUP)

□ Faculty of Fine Arts (FBAUP)

□ Faculty of Sciences (FCUP)

□ Faculty of Nutrition and Food Science (FCNAUP)

□ Faculty of Sports (FADEUP)

□ Faculty of Law (FDUP)

□ Faculty of Economics (FEP)

□ Faculty of Engineering (FEUP)

□ Faculty of Pharmacy (FFUP)

□ Faculty of Arts (FLUP)

□ Faculty of Medicine (FMUP)

□ Faculty of Dental Medicine (FMDUP)

□ Faculty of Psychology and Educational Science (FPCEUP)

□ Abel Salazar Institute of Biomedical Sciences (ICBAS)

**ASPECTS RELATED TO SARS-CoV-2 INFECTION**

**P5. Since the beginning of the year (2020), have you been under active surveillance by the health authority for having been in close contact with a suspected or confirmed case of infection by the new coronavirus?**

- No
- Yes

**P6. Since the beginning of the year (2020), have you personally been in contact with anyone with a confirmed diagnosis of infection by the new coronavirus before they were considered to have recovered?**

- No
- Yes

**P7. Since the beginning of the year (2020), have you had any symptom unusual and/or all of a sudden (like cough, fever, vomiting, or others?)**

- No
- Yes

If yes:

**P7.1** Which one(s)?

| **Respiratory symptoms** |  |  | Are you symtomatic? | |
| --- | --- | --- | --- | --- |
| Persistent cough | ☐ Y | ☐ N | ☐ Y | ☐ N |
| Respiratory difficulties (Dyspnea) | ☐ Y | ☐ N | ☐ Y | ☐ N |
| Sore throat (Odynophagia) | ☐ Y | ☐ N | ☐ Y | ☐ N |
| **Gastrointestinal symptoms:** |  |  |  |  |
| Vomiting/Nausea | ☐ Y | ☐ N | ☐ Y | ☐ N |
| Diarrhea | ☐ Y | ☐ N | ☐ Y | ☐ N |
| **Other symptoms:** |  |  |  |  |
| Fever (>37.5ºC) | ☐ Y | ☐ N | ☐ Y | ☐ N |
| Chills and shivering | ☐ Y | ☐ N | ☐ Y | ☐ N |
| Headache (Cephalalgia) | ☐ Y | ☐ N | ☐ Y | ☐ N |
| Joint pain (Arthralgia) | ☐ Y | ☐ N | ☐ Y | ☐ N |
| Muscle pain throughout the body (Myalgia) | ☐ Y | ☐ N | ☐ Y | ☐ N |
| Loss of smell (Anosmia) | ☐ Y | ☐ N | ☐ Y | ☐ N |
| Loss of taste (Dysgeusia) | ☐ Y | ☐ N | ☐ Y | ☐ N |
| General weakness or asthenia | ☐ Y | ☐ N | ☐ Y | ☐ N |
| Other 1 : __________________ | ☐ Y | ☐ N | ☐ Y | ☐ N |
| Other 2 : __________________ | ☐ Y | ☐ N | ☐ Y | ☐ N |
| Other 3 : __________________ | ☐ Y | ☐ N | ☐ Y | ☐ N |

**P8. Have you ever been tested for SARS-CoV-2 infection?**

(consider only the diagnostic tests)

- No
- Yes

If yes:

**P8.1.** Did you receive a confirmed diagnosis of SARS-CoV-2 infection?

- - No
  - Yes

If yes:

**P8.1.1.** On what date did you have a positive diagnosis?

|___|___| - |___|___| - |___|___|___|___|

**P8.1.2.** On what date were you considered to have recovered?

|___|___| - |___|___| - |___|___|___|___|

**P9.** [If never been tested or diagnosed ] **From your point of view, what is the probability that you have already been infected with SARS-CoV-2 (virus that causes COVID-19)??**

- Very low
- Low
- Moderate
- High
- Very high

**PREVENTIVE MEASURES**

**P10. Overall, would you say that you contact daily with how many people in the following age groups within 2 meters over 15 minutes?**

(consider everyone including people in the household and regardless of mask use)

**P10.1.** less than 18 years of age: ____ (number)

**P10.2.** between 18 and 64 years of age: ____ (number)

**P10.3.** 65 years or more: ___ (number)

**P11. On a daily basis how often you use the following types of masks?**

| **Non-surgical** | - Never | - Rarely | - Often | - Always |
| --- | --- | --- | --- | --- |
| **Surgical** | - Never | - Rarely | - Often | - Always |
| **Respirators** | - Never | - Rarely | - Often | - Always |

**P3. At the University premises how often do you use the following types of masks?**

| **Non-surgical** | - Never | - Rarely | - Often | - Always |
| --- | --- | --- | --- | --- |
| **Surgical** | - Never | - Rarely | - Often | - Always |
| **Respirators** | - Never | - Rarely | - Often | - Always |

**P12.Overall, how often do you wear a mask:**

| **Outdoors** | - Never | - Rarely | - Often | - Always |
| --- | --- | --- | --- | --- |
| **In enclosed spaces, including public transport** | - Never | - Rarely | - Often | - Always |
| **At home** | - Never | - Rarely | - Often | - Always |

**P13. Para a higiene das mãos usa, com que frequência usa:**

**At home:**

| **Alcohol-based antiseptic solution** | - Never | - Rarely | - Often | - Always |
| --- | --- | --- | --- | --- |
| **Water and soap** | - Never | - Rarely | - Often | - Always |

**At the University premises :**

| **Alcohol-based antiseptic solution** | - Never | - Rarely | - Often | - Always |
| --- | --- | --- | --- | --- |
| **Water and soap** | - Never | - Rarely | - Often | - Always |

**Other places:**

| **Alcohol-based antiseptic solution** | - Never | - Rarely | - Often | - Always |
| --- | --- | --- | --- | --- |
| **Water and soap** | - Never | - Rarely | - Often | - Always |

**P14. In general, how many time do you wash your hands daily:**

- 0-1
- 2-5
- 6-10
- More than ten

**RESULTS OF SARS-CoV-2 SCREENING**

**P15: Was the test done?**

- No
- Yes

If not:

**P15.1.** For what reason?

- - Refused the test
  - Other. Which reason? _______________________________________________

If yes:

**P16. Brand:**

|  | **Lot:** | **Expiry date:** |
| --- | --- | --- |
| - *STANDARD Q COVID-19 IgM/IgG Combo Test* | - QCO502004B/Sub B-1 | - 08/01/2021 |
| - Other ______________________ | - Other | - Other |

**P17. Result**

- Invalid
- valid

If valid:

**P17.1.** Result:

| **IgM** | - Reactive | - Non-reactive | - Inconclusive |
| --- | --- | --- | --- |
| **IgG** | - Reactive | - Non-reactive | - Inconclusive |

If invalid or inconclusive:

**P17.2.** Do you accept to repeat the test?

□ No

□ Yes

If yes:

**P17.2.1. Brand:**

|  | **Lot:** | **Expiry date:** |
| --- | --- | --- |
| - *STANDARD Q COVID-19 IgM/IgG Combo Test* | - QCO502004B/Sub B-1 | - 08/01/2021 |
| - Other ______________________ | - Other | - Other |

**P17.2.2.** Repeat result:

- - - Invalid
    - valid

If valid:

**P17.1.** Result:

| **IgM** | - Reactive | - Non-reactive | - Inconclusive |
| --- | --- | --- | --- |
| **IgG** | - Reactive | - Non-reactive | - Inconclusive |

**Observations:**

|  |
| --- |
